# Enhancing Stress Detection: A Comprehensive Approach through rPPG Analysis and Deep Learning Techniques

**DOI:** 10.3390/s24041096

**Published:** 2024-02-07

**Authors:** Laura Fontes, Pedro Machado, Doratha Vinkemeier, Salisu Yahaya, Jordan J. Bird, Isibor Kennedy Ihianle

**Affiliations:** Department of Computer Science, Nottingham Trent University, Nottingham NG1 4FQ, UK; n1119003@my.ntu.ac.uk (L.F.); pedro.machado@ntu.ac.uk (P.M.); doratha.vinkemeier@ntu.ac.uk (D.V.); salisu.yahaya@ntu.ac.uk (S.Y.); jordan.bird@ntu.ac.uk (J.J.B.)

**Keywords:** 1D Convolutional Neural Network (1D-CNN), Deep Learning (DL), Gated Recurrent Units (GRU), Long Short-Term Memory (LSTM), physiological signals, remote photoplethysmography (rPPG), stress detection

## Abstract

Stress has emerged as a major concern in modern society, significantly impacting human health and well-being. Statistical evidence underscores the extensive social influence of stress, especially in terms of work-related stress and associated healthcare costs. This paper addresses the critical need for accurate stress detection, emphasising its far-reaching effects on health and social dynamics. Focusing on remote stress monitoring, it proposes an efficient deep learning approach for stress detection from facial videos. In contrast to the research on wearable devices, this paper proposes novel Hybrid Deep Learning (DL) networks for stress detection based on remote photoplethysmography (rPPG), employing (Long Short-Term Memory (LSTM), Gated Recurrent Units (GRU), 1D Convolutional Neural Network (1D-CNN)) models with hyperparameter optimisation and augmentation techniques to enhance performance. The proposed approach yields a substantial improvement in accuracy and efficiency in stress detection, achieving up to 95.83% accuracy with the UBFC-Phys dataset while maintaining excellent computational efficiency. The experimental results demonstrate the effectiveness of the proposed Hybrid DL models for rPPG-based-stress detection.

## 1. Introduction

Stress in humans is related to mental health and well-being [1]. It is the biological response to a situation such as a threat, challenge, or physical and psychological barrier [2]. The sympathetic nervous system (SNS) and the parasympathetic nervous system (PNS) are two components of the autonomic nervous system (ANS) that directly affect how the body reacts to stress [3,4]. In highly stressful events, the SNS executes the *fight or flight* survival response. As a result, the body redirects its efforts toward fighting off threats. Given its subjective nature, identifying and monitoring the onset, duration, and severity of stressful events is challenging. This is especially true in workplace situations [5] where there is often an intelligent choice to ignore stress for professional gain. Recent studies have shown an increase in stress levels in the office environment [6]. Due to the plasticity of the brain, chronic or persistent stress has been shown to increase the volume of the amygdala, a structure within the limbic system that defines and regulates emotions, stores emotional memories, and, most importantly, executes the fight or flight response [7]. Similarly, chronic stress is associated with a reduction in the mass of the prefrontal cortex [8], which is used to intelligently regulate thoughts, actions, and emotions.

Recent research in the field has introduced various sensor-based solutions for stress detection, as evidenced by studies such as [4,9,10]. Although some of these solutions use only a single type of sensor, others employ multimodal sensing. Traditionally, electrocardiography (ECG) has been used to measure heart rate variability (HRV) for stress detection [11]. Biomarkers like galvanic skin response (GSR), electrodermal activity (EDA), respiration, and electromyography (EMG) are increasingly recognized for assessing affective states and stress levels [12,13,14], utilising sensing devices. While these traditional sensor types are considered the gold standard and provide excellent opportunities for the measurement of stress-related biomarkers, the ease of use for these devices in a practical scenario becomes a challenge, as experimentation can only be carried out in a designated equipped setting. The focus of research is shifting to developing simpler and more convenient sensing solutions that are applicable to everyday life to measure physiological parameters. Recent advances in technology have led to significant developments in wearable and personal sensing devices with applications in healthcare, for example, the use of a wearable device to capture physiological data for health monitoring [15,16,17,18,19,20]. These devices include chest bands [15,16,21,22], portable ECG devices [17,23], etc. HRV parameters can be measured using wristbands such as Empatica E4 wristband [18,24], Microsoft Band 2 [19,25], Polar watch [20,26], and Fitbit watch [20,26], among others. Researchers analyse personal data from these devices to provide relevant insights into the individual’s physical and health status. Although these devices show promise and provide a non-intrusive means of acquiring data for stress detection models, a major limitation of these devices relates to the size, making them uncomfortable for practical use cases [27].

On the contrary, rPPG technology measures Blood Volume Pulse (BVP) using a camera, eliminating the need for sensor attachments [28,29]. By extracting skin pixels from facial data captured by the camera, rPPG technology utilises changes in skin colour corresponding to heartbeat to obtain the BVP signal [28,30,31,32]. This method simplifies the measurement, reduces sensor complexity, and avoids attachment-related problems. Furthermore, rPPG can be used to capture HRV measures for analysis, especially in healthcare applications. The widespread availability of cameras in the form of webcams or smartphones makes rPPG technology easily accessible to anyone. Due to its advantages, rPPG finds applications in healthcare, fitness, and forensic science. Integration of rPPG technology into smart mirrors or smartphones increases its potential as a professional health indicator. Although still in an early stage, rPPG-based non-contact affective computing has become a growing area of research in recent years, which can drastically improve human–computer interaction in real time for stress detection. This paper explores the feasibility of end-to-end methods for recognising stress by proposing a rPPG-based stress detection system to leverage non-contact and physiological techniques, facilitating the continuous monitoring of pervasive long-term biomedical signals. The contributions made in this paper are as follows:

- A novel system leveraging non-contact and physiological techniques is proposed, enabling the continuous monitoring of pervasive biomedical signals for long-term stress detection.
- Hybrid DL networks and models for rPPG signal reconstruction and Heart Rate (HR) estimation to significantly improve accuracy and efficiency in stress detection up to 95.83% with the UBFC-Phys dataset.
- Extensive experiments and empirical evaluations of Deep Learning (DL) models for stress detection provide valuable insights and comparisons.

The remainder of this paper is structured as follows. Section 2 presents a comprehensive literature review of the existing approaches, while Section 3 introduces the methodology, collection protocol, and preprocessing steps. In Section 4, the experimental results are discussed while the conclusion and future work plan are outlined in Section 5.

## 2. Related Work

The term *stress* was initially introduced into medical terminology in 1936, referred to as a syndrome produced by diverse nocuous agents that seriously threaten homeostasis [33]. Selye’s experiments demonstrated that prolonged exposure to severe stress could lead to disease and tissue damage [34]. Recently, research on stress, its causes, and implications has gained traction [4,9,10,12,13,14]. It is characterised by a complex interactive phenomenon, arising when a situation is deemed important, carries the possibility of damage, and requires psychological, physiological, and/or behavioural actions [4,9,10]. Understanding stress involves distinguishing between stressors, stress responses, and stress biomarkers. Stressors are stimuli that disrupt normal activity, stress responses are symptoms triggered by stressors, and biomarkers reflect interactions between a biological system and potential hazards [3,4,9,10]. The human body responds to stressors through mechanisms such as the hypothalamic–pituitary–adrenal (HPA) axis, ANS, and the immune system [35]. The HPA axis releases hormones, including cortisol, in response to stressors, initiating the “fight or flight response”, leading to physiological reactions from the ANS, increasing SNS activity, and decreasing PNS activity [3,4]. Cortisol levels and other physiological measures such as body temperature, respiration rate, pulse rate, HRV, and blood pressure (BP) have been identified as standard stress biomarkers [15,16,17,21,22,23]. Several methods for stress detection include questionnaires, ECG, electroencephalogram (EEG), BP using arm cuff, sampling saliva cortisol and other biomarkers from blood tests [36,37,38]. Self-reporting tools such as the Perceived Stress Scale and Depression Anxiety Stress Scale are widely used to measure perceived stress, but have limitations such as biased responses and subjectivity [39]. ECG measures changes in heart rhythm due to emotional experiences; providing information about HRV usually requires a visit to a medical facility. EEG captures electrical signals in the brain, correlating brain waves (beta and alpha) to stress, but conventional EEG machines are impractical for managing daily stress [40,41]. Biomarkers such as cortisol in salivary and hair samples are associated with chronic stress but are invasive and time-consuming. Blood pressure measured with a sphygmomanometer is accurate, but requires a trained professional [36,37,38]. Ambulatory Blood Pressure Measurement (ABPM) devices offer home monitoring, but lack widespread validation and can be influenced by factors other than stress [42]. While traditional sensor types are acknowledged as the gold standard, offering excellent opportunities for measuring stress-related biomarkers, their practical use in everyday situations poses a significant challenge. Emerging technologies have focused on developing simpler and more convenient sensing solutions applicable to daily life to measure physiological biomarkers. Wearable and personal sensing devices, such as chest bands, wrist bracelets, and portable ECG devices [15,18,21,24], have played a pivotal role in this evolution.

Conventional approaches to stress detection have drawbacks that are not in line with modern lifestyles and real-time monitoring. These methods are invasive, prone to bias, incur substantial costs, and require time-consuming travel to clinical settings. Over the past two decades, there has been a noticeable shift towards technology-driven approaches for more efficient, cost-effective, and less intrusive stress measurement compatible with modern lifestyles. Wearable devices, mobile applications, and Machine Learning (ML) algorithms have revolutionised stress detection and measurement. One approach is measuring HRV using wearable devices such as smartwatches, fitness trackers, and chest straps, allowing continuous and long-term monitoring of stress levels [16,17,20,23,26]. Typically, as HRV measures are inherently nonlinear, ML algorithms and other statistical data-driven methods such as Modified Varying Index Coefficient Autoregression Model (MVICAR) [43] can be applied in stress detection systems. ML algorithms have enabled accurate and efficient HRV-based stress detection and classification systems [29,44,45,46,47]. EDA, which measures the electrical activity of sweat glands, is another method that can be monitored with wearable devices, providing continuous and real-time monitoring of stress levels. Mobile applications using EDA-based biofeedback help individuals manage stress by providing real-time feedback and stress reduction techniques [16,25]. However, EDA measurement is sensitive to environmental factors, skin conditions, and medications, affecting the precision.

The COVID-19 pandemic has stimulated interest in remote healthcare, leading to research using cameras for the estimation of rPPG signals and real-time monitoring, addressing the need for non-invasive, contactless, and accessible methods for stress assessment [48,49]. rPPG offers a non-invasive means of measuring BVP remotely. This approach requires only a camera and an ambient light source. With this, HRV measures, pulse rate, and breathing rate can be measured using an everyday camera for facial video analysis to remotely detect and monitor stress [28,30,31,32]. There have been a growing number of research papers. For example, Benezeth et al. [46] proposed an rPPG-based algorithm that estimates HRV using a simple camera, showing a strong correlation between the HRV features and different emotional states. Similarly, Sabour et al. [29] proposed an rPPG-based stress estimation system with an accuracy of 85.48%. Some other works on the use of rPPG are encouraging, indicating that noncontact measures of some human physiological parameters (e.g., breathing rate (BR) and Heart Rate (HR)) are promising and have great potential for various applications, such as health monitoring [47,50] and affective computing [51,52,53]. While these contributions are noteworthy, this paper significantly advances the field by introducing Hybrid Deep Learning (DL) networks and models for rPPG signal reconstruction and Heart Rate (HR) estimation. This novel approach presents a substantial improvement in accuracy and efficiency in stress detection, achieving up to 95.83% accuracy with the UBFC-Phys dataset. The integration of Hybrid DL networks represents a contribution, offering enhanced capabilities for signal reconstruction and stress classification. Considering these, rPPG is well-suited for both business and everyday applications and has the significant advantage of measuring ECG and photoplethysmography (PPG).

Wearable and contactless devices offer promising alternatives for stress measurement, providing convenient and non-invasive methods for continuous monitoring. However, the quality and accuracy of the data generated by these devices can vary. A major limitation to adapting rPPG is evident in the decrease in the signal-to-noise ratio, which requires advanced signal processing. Many articles lack peer review and validation in clinical settings, raising concerns about the reliability of data. Although wearable devices can be sensitive to factors such as movement, heat, and transpiration, leading to inaccurate measurements, ease of use, especially during sleep or physical activities, is another huge limitation. Individuals with skin sensitivities, allergies, or specific health conditions may also find wearing these devices intolerable.

## 3. Method

**Figure 1:**
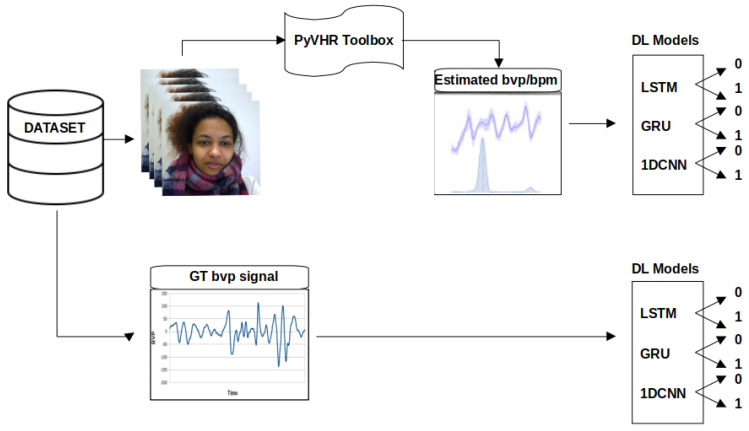
Stress detection framework. The video frames serve as inputs to the pyVHR toolbox, enabling the extraction of rPPG BPM signals from facial regions within the frames. The derived BPM signals are subsequently channelled through DL models (LSTM, GRU, and 1D-CNN), culminating in stress classification outcomes.

The proposed methodology consists of three main parts, as shown in Figure 1. The primary objective is to detect social stress using contactless physiological signals extracted from facial videos through DL techniques. In the first part, a pyVHR toolbox (Python framework for Virtual Heart Rate) [54] is used to capture and estimate the beats per minute (BPM) from facial video data. The second part involves the increase in the estimated BPM and is subsequently input into four DL models (Recurrent Neural Network (RNN), LSTM, GRU, and 1D-CNN). The performance of these models is then evaluated and compared on the basis of specific metrics. The proposed methodology is implemented using Python 3 and relevant libraries for data manipulation, leveraging an NVIDIA graphics processing unit (GPU) with Compute Unified Device Architecture (CUDA) version 12.2 and CUDA Deep Neural Network (CuDNN) library. It should be noted that the default parameters of pyVHR, including a window size of 8, patch size of 40, and pre/post filter, were used for the estimation of BPM. The selected methods include Regions of Interest (ROI) approaches: holistic and convex hull, as well as CuPy CHROM, Torch CHROM, and CuPy POS. Refer to Table 1 for a brief overview of the methods.

### 3.1. Dataset and Data Processing

**Figure 2:**
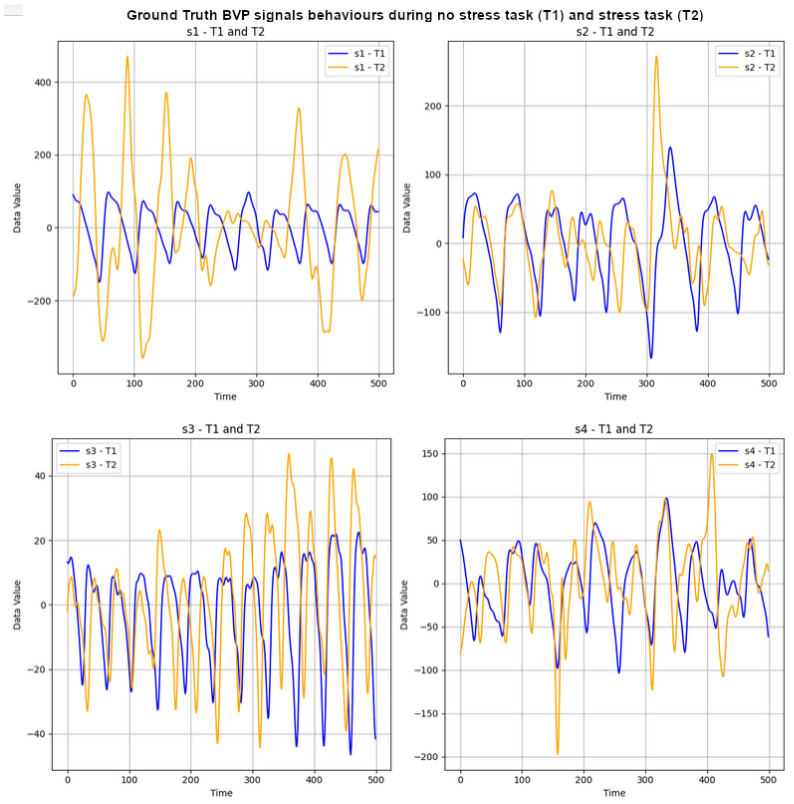
GT BVP signals behaviour during no-stress task (T1) and stress task (T2) of subjects s1 to s4.

The UBFC-Phys dataset includes data from 56 healthy subjects, with 12 participants excluded due to technical and consent issues [29]. The participants, aged between 19 and 38 (mean age 21.8, standard deviation 3.11), comprise 46 women and 10 men. In the study, stress levels were induced using a modified version of the Trier Social Stress Test (TSST) [55]. The participants completed three tasks: a 10-minute rest task serving as a baseline, a speech task, and an arithmetic task. Speech and arithmetic tasks aimed to induce stress through a social evaluation threat. In the test scenario, the speech task simulated a job interview, introducing an additional expert via video call to enhance social-evaluative threat. The arithmetic task involved a countdown with variations. For the purposes outlined in this paper, attention is given to ground-truth (GT) BVP signals labelled as T1 and T2 for the stress and non-stress classes, respectively. These signals, obtained using the Empatica 4 wristband at a 64 Hz sampling rate, consist of vectors with 11,520 data points each (64 × 180 = 11,520). Subsequently, the first 500 data points of the GT BVP signals for subjects s1 to s4 were plotted to visually depict the impact of stress (T1) and non-stress (T2) on signal behaviour. Refer to Figure 2 for these graphs.

Data processing included the application of the Fast Fourier Transform (Fast Fourier Transform (FFT)) to generate frequency domain features from the Blood Volume Pulse (BVP) signals. In addition, the data augmentation was implemented with Linear Interpolation and Gaussian White Noise.

Linear interpolation, as illustrated by Equation (1), augments by estimating values between existing data points, creating straight lines connecting these points.

(1) $$y=y_{1}+\left(x-x_{1}\right)\frac{y_{2}-y_{1}}{x_{2}-x_{1}}$$

where $x_{1}$ and $y_{1}$ are the first coordinates, $x_{2}$ and $y_{2}$ are the second coordinates, *x* is the point to perform the interpolation, and *y* is the interpolated value.

Alternatively, the Gaussian White Noise augmentation method generates series of random values using the Gaussian distribution; see Equation (2) below. The resulting sequence exhibits white noise characteristics. Gaussian White Noise serves multiple purposes beyond dataset expansion. It is valuable to simulate uncertainty, randomness, or inherent variability present in real-world data.

(2) $$series\left[i\right]=X_{i},fori=1,2,3,\ldots ,1000$$

where $X_{1}$, $X_{2}$, $X_{3}$, …, $X_{1000}$ are independent and identically distributed random variables following a Gaussian distribution with mean μ=0.0 and standard deviation σ=1.0.

### 3.2. Deep Learning Models

**Figure 3:**
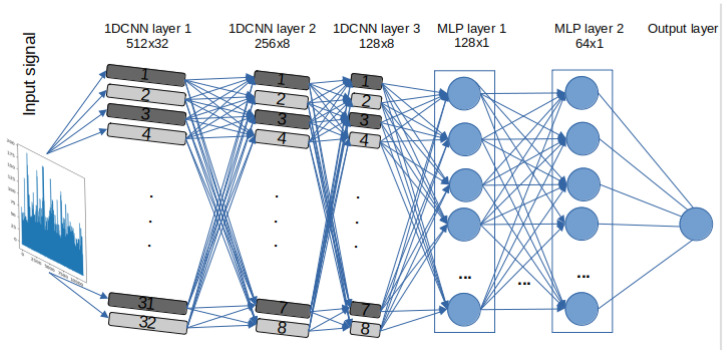
1D 3x CNN-2x MLP architecture—labelled 1D-CNNv2.

A set of DL models are selected to detect stress and evaluate the effectiveness and efficiency of the models. Due to intrinsic structural differences between DL models based on RNN, specifically LSTM and GRU, and Convolutional Neural Networks (CNN), three 1D-CNN-Multilayer Perceptron (MLP) models were designed. One of these models closely mirrors the architectures of RNN-based models in terms of the number of neurons, represented as “filters” in CNNs. However, instead of utilising LSTM or GRU layers, Convolutional One-Dimensional (Conv1D) layers were used. These models also include Maxpooling1D layers and flatten layers, along with specific parameters and functions, such as kernel size and Rectified Linear Unit (ReLU) activation. The other two 1D-CNN models have additional CNN and MLP layers and different “pool size”. It is important to note that the limited sample size of estimated BPM signals (only 172 data points per video) from the pyVHR toolbox prevented the evaluation of the performance of 1D-CNN models versions 2 and 3, given their respective architectures. For a detailed architecture, layer descriptions, parameters, and functions of the 1D-CNN-MLP models, please refer to Table 2. The design flow of the 1D-CNN with 3 CNN and 2 MLP layers, labelled “ CNNv2”, is illustrated in Figure 3.

### 3.3. Performance Evaluation

The metrics chosen to evaluate the models needed to be suitable for the classification of categorical variables ”stress” and ”no-stress”. For that reason, the metrics Accuracy-Ac, Recall-Re, Precision-Precision (Pr), and F1-Score (F1) were selected. Each of these metrics assesses the models’ classification performance from a different perspective.

**Accuracy**—It provides a general sense of how well the model is performing between stress and non-stress classification. The higher the value, the greater the model’s accuracy.

(3) $$Ac=\frac{TP+TN}{TP+FN+TN+FN}$$

**Recall**—This metric is also known as sensitivity metric, or true positive rate. It computes the proportion of true positive predictions out of all actual positive instances. In the context of this research project, a high recall value indicates that the model is sensitive to detecting social stress, which is critical for its practical application.

(4) $$Re=\frac{TP}{TP+FN}$$

**Precision**—Calculates the proportion of true positive predictions out of all positive instances. The higher the value, the more accurate the model is predicting the true positive instances. This helps minimise false positives, which is crucial when dealing with stress assessment.

(5) $$Pr=\frac{TP}{TP+FP}$$

**F1**—This metric provides a balanced view of the model’s performance by considering both precision and recall. In stress classification, achieving a balance between minimising false positives Pr and false negatives Re is vital. A high F1 indicates that the model accurately identifies instances of social stress and minimises false classifications.

(6) $$F1=2\times \frac{Pr\times Re}{Pr+Re}$$

where the classification outcomes are *True Positive (TP)*, *True Negative (TN)*, *False Positive (FP)*, and *False Negative (FN)*.

## 4. Experimental Results

**Figure 4:**
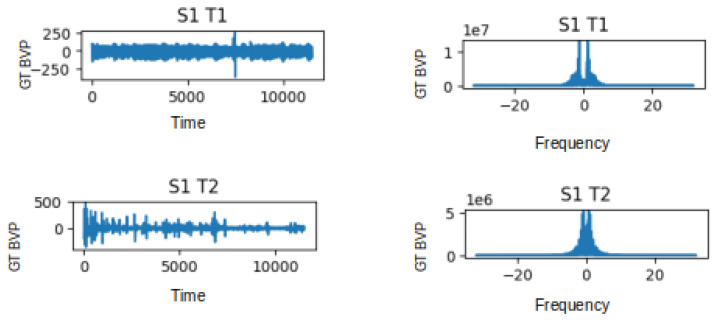
Graphs depicting the Time Domain (TD) and Frequency Domain (FD) representations of the GT BVP signals for subject 1 during tasks T1 and T2.

The visualisations provided in Figure 4 offer a distinct view of the contrasting characteristics between the non-stress task (T1) and the stress-induced task (T2) in both the time and frequency domains. In the time domain analysis, the T1 signal exhibits fluctuations within the range of −250 to 250 units, while in the presence of stress during T2, this range becomes wider, spanning from −500 to 500 units. This change in range suggests a potentially heightened physiological response during the stress task. Likewise, when we delve into the frequency domain, we notice a parallel pattern. In the frequency domain representation, the T1 signal presents values oscillating between 0 and 1, whereas the T2 signal exhibits a wider span of 0 to 5. This expanded variation in the frequency domain further emphasises the distinction between the non-stress and stress-induced states. Moreover, the implications of these observations extend beyond mere visualisation. The frequency domain signal has immense potential as a feature for training and testing deep learning methods aimed at stress classification. While the raw BVP signal encapsulates temporal patterns, the frequency domain offers insight into the underlying frequency components that contribute to those patterns. By extracting features from the frequency domain, deep learning models can potentially capture and leverage distinctive spectral characteristics related to stress. The plots in Figure 4 illustrate the GT BVP signals of subject 1 during tasks T1 and T2 before and after FFT being applied to the data.

**Figure 5:**
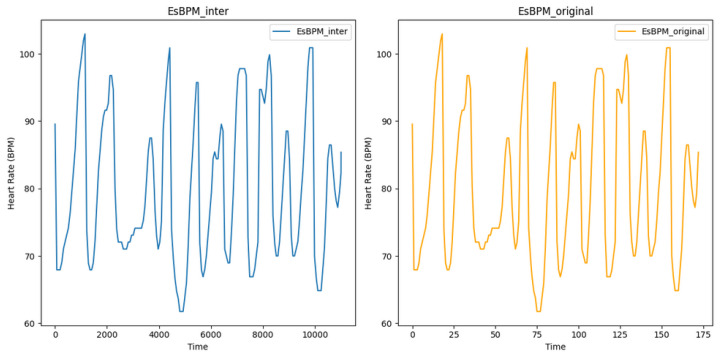
Plot of estimated BPM extracted from video T1 of subject 1, using the method CuPy CHROM, before and after augmentation using linear interpolation.

Figure 5 shows the estimated heart rate (BPM) extracted from video T1 of subject 1, using the CuPy CHROM method from the pyVHR toolbox. This visualisation illustrates the state before and after augmentation using linear interpolation, where it is possible to infer that expanding the original dataset of 173 data points to 11,009 data points did not alter the underlying signal, reinforcing the consistency between the original and augmented data. The processed and augmented dataset is then partitioned into training, validation, and test datasets using 10% for validation and 10% for testing.

**Figure 6:**
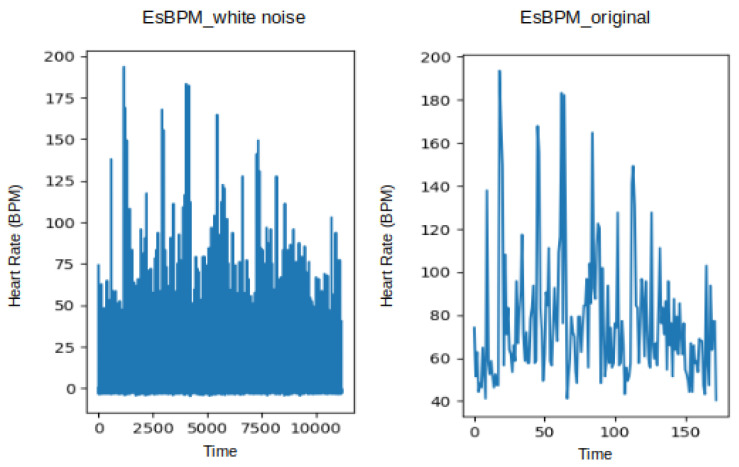
Plot of estimated BPM extracted from videos T1 of subject 1, using the method CuPy CHROM, before and after augmentation using white noise.

Likewise, Figure 6 shows the estimated heart rate (BPM) plotted from the T1 and T2 videos of subject 1, using the CuPy CHROM method from the pyVHR toolbox. This visualisation illustrates the state before and after augmentation using white noise, where it is possible to infer that expanding the original dataset of 173 data points to 11,180 data points did not alter the underlying signal.

### 4.1. Classification Results

Three distinct DL methods (LSTM, GRU, 1D-CNN), each with different architectures (as detailed in Table 2), were implemented to identify the optimal model to effectively classify stress levels. Although this work focuses on building the best DL model to accurately classify stress status by extracting rPPG from face videos, this classification task was conducted using both GT-BVP signals computed from videos of the UBFC-Phys dataset separately in order to compare the performance of the DL models on the GT-BVP and the rPPG.

#### 4.1.1. Performance Analysis of the DL Methods Applied to the GT Signal

**Figure 7:**
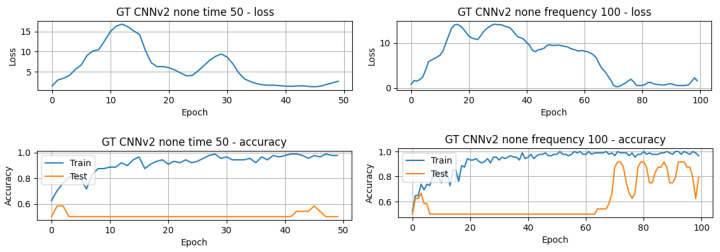
Validation loss and train and accuracy curves of the GT-1D-CNNv2 model.

The results in Table 3 present the top-performing results achieved in this article for both the raw GT (TD) data and the processed GT (FD) data. The results are arranged in descending order, highlighting the best-performing models and their respective accuracy. Additionally, the computation time for each model is also provided to allow for comparison of the execution times of the different models. There is a noticeable difference in computational efficiency between the CNN models and the LSTM and GRU models. The 1D-CNNv1 model completed 50 epochs in just 4.24 s, while the LSTMv2 model required approximately 1 min and 30 s to achieve the same. The accuracy of the models varies between approximately 41.67% and 83.33%, and it is obvious that the best results were obtained using the TD data. However, some models exhibit different performances depending on the domain. For example, the 1D-CNNv2 model achieves significantly better accuracy (83.33%) in the time domain compared to its accuracy (50.00%) in the frequency domain. On the contrary, the GRUv2 model demonstrates a higher accuracy (62.50%) in the FD compared to its accuracy (58.33%) in the TD. Concerning the number of epochs for training and testing the models, it is possible to infer that the majority of the models only needed 50 or fewer epochs. On the other hand, the 1D-CNNv2 model achieved its higher performance around the 60th epoch, as can be seen in Figure 7.

Regarding the precision and recall in Table 3, precision in some cases is balanced with recall, while in others, trade-offs are evident. As previously mentioned, models with both high precision and high recall scores are effective at correctly classifying stress instances (true positives) and minimising both false positives and false negatives. For instance, the 1D-CNNv2 model achieved this balance, with an accuracy of 83.33% and Precision and Recall of 83.33%. On the other hand, models with high Recall, but lower Precision predict more instances as stressed, including those that are uncertain. This is useful when capturing all stress instances is a priority, even if it means more false positives. The GRUv1 model in the FD shows this pattern, with Recall of 91.67% but Precision of 61.11%. It is also clear that the **1D-CNNv2** model achieved the highest accuracy **83.33%** among the tested methods. This suggests that it might be the most effective model for classifying stress and non-stress states from the GT-BVP signals. From Table 4, it can be inferred that the results achieved by the traditional machine learning method employed by the dataset’s authors **75%** and the CNN-MLP model utilised in the study by Hasanpoor et al. **82%** [54] were both exceeded in this work **83.33%**.

#### 4.1.2. Performance Analysis of the DL Methods Applied to the rPPG Signal

Moving forward to the performance of the DL models on the estimated BPM, these were obtained considering the different methods of BPM extraction on the pyVHR toolbox (CuPy-CHROM, CuPy-POS, and Torch-CHROM), different epochs (50–100), augmentation techniques (none, linear interpolation, and white noise), DL model versions, input domains (TD and FD), evaluation metrics (accuracy, precision, recall, and F1 score), and execution times. The training and testing generated over two hundred lines of results. The best results per DL model version and per pyVHR method are depicted in Table 5. With regard to these results, several conclusions can be drawn from this table. On a wider perspective, the accuracy ranges from 79.17% to 95.83%, indicating the DL models’ effectiveness in distinguishing between stress and non-stress states, which in the opinion of the authors can be considered a very good performance across the models. Precision and recall values vary across all models, with some achieving 100% and others slightly lower (the lowest being 73.33%), and the F1 score follows the same trend. Considering the time domains and augmentation techniques, it is possible to infer that the majority of the models excelled in the frequency domain, whereas the 1D-CNNv3 demonstrated high scores across all metrics in TD. In terms of augmentation techniques, it is possible to infer that interpolation and no additional augmentation achieved the best performances across all models. Furthermore, both CuPy-CHROM and Torch-CHROM pyVHR methods can be a good choice for estimating BPM from facial videos for stress classification, because all three CNN models achieved higher performances, although with distinctive augmentation techniques and domains. Regarding the train and test times, these range from few seconds to over two minutes, with CNN having the best execution times compared with the LSTM and the GRU models.

**Figure 8:**
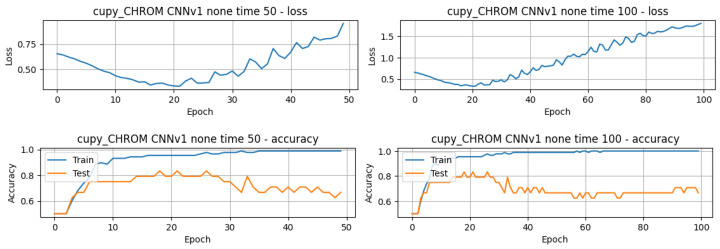
Plot of estimated BPM extracted from videos T1 of subject 1, using the method CuPy CHROM, before and after augmentation using white noise.

In terms of the number of epochs for training and testing, it is possible to infer that, for the great majority of the models, less than 50 epochs were needed to train and test the model, with a few exceptions, as in the case of the model that achieved the best overall performance, 1D-CNNv1 with the configuration white noise and FD, whose performance slightly improved from around 91.70% to 95.83%. The validation loss and accuracy curves also reflect that difference, where it can be seen that the model’s performance slightly improved after around 60 epochs, with an increase in the testing curve and a decrease in the loss curve (refer to Figure 8).

Considering the importance of accuracy, precision, and recall metrics, along with the focus on real-world deployment utilising edge devices, the following models appear to be the stronger candidates: **1D-CNN models**, namely **1D-CNNv1**, using the CuPy-CHROM method, white noise augmentation, FD, and 100 epochs, with a mere 7.8 s of execution time; 1D-CNNv2, also using the CuPy-CHROM method, with linear interpolation augmentation, FD, and 50 epochs; and the 1D-CNNv3 using the Torch-CHROM method, with linear interpolation augmentation, TD, and 50 epochs.

**Figure 9:**
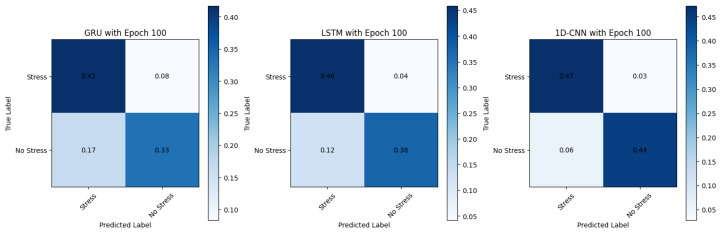
Confusion matrix showing performance across different models.

These models, as illustrated in the normalised confusion matrix in Figure 9, consistently achieve high accuracy **95.83%**, precision, and other metrics across TD, FD, and pyVHR methods. They are well-suited for real-time applications due to their relatively lower training times compared to the LSTM and the GRU models. Furthermore, these models demonstrate that they are efficient in processing sequential data like time series, making them suitable for processing heart rate data extracted from videos. Moreover, the balanced precision and recall they offer make them well-suited for stress and non-stress classification, as avoiding false positives and false negatives is crucial.

As shown in Table 6, two of the three CNN models (1D-CNNv2 and 1D-CNNv3) achieved perfect scores (100%) in all performance metrics. These results were omitted from the best results in Table 5 and are likely the consequence of overfitting, due to training a heavy model on a small dataset. The authors believe that it is reasonable to assume that the deployment of these models, along with their associated weights, to real-world data scenarios would probably yield performance outcomes that are less impressive.

## 5. Conclusions and Future Work

This paper has successfully established a robust framework for remote stress detection through the analysis of physiological signals derived from facial videos. The primary goal was to ascertain an advanced DL model for stress classification, surpassing the capabilities of traditional ML techniques. The adoption of three DL methods (LSTM, GRU, and CNN) and their refinement through empirical optimization yielded significant achievements, including an impressive 95.83% accuracy in classifying stress from rPPG signals. The outstanding computational efficiency of the best-performing DL model, 1D-CNNv1, aligns seamlessly with the prospect of deploying the framework on edge devices. The exploration of augmentation techniques, particularly linear interpolation and the absence of augmentation, showcased promising outcomes, highlighting their efficacy in enhancing model performance. The proposed methodology holds significant potential to influence stress-related policies, practices, and management, potentially fostering increased user engagement with stress detection tools. However, it is crucial to acknowledge a major limitation inherent in the rPPG approach, centered around privacy concerns stemming from the utilisation of cameras and the diversity of the participants. The privacy issue emphasises the need for user consent and necessitates a careful balance between the potential advantages of the approach and the preservation of individual privacy rights. It is imperative to underscore that the rich insights provided by this approach should be accompanied by stringent privacy measures, ensuring that user consent is sought and respected throughout the stress detection process. Future work will focus on improving signal extraction through alternative physiological sensing tools and optimising parameters in existing toolboxes. Exploring additional augmentation techniques and advancing DL methods, particularly focusing on 1D-CNN, stand as promising paths for further enhancement. Rigorous validation through cross-validation and testing on diverse datasets is paramount to assess model robustness and ensure generalisation across various scenarios. Furthermore, future investigations could also consider the potential influence of participant ethnicity on model accuracy, recognising the importance of addressing diversity in the dataset and its implications for the broader applicability of the stress detection framework.

## Figures and Tables

**Table 1:** Parameters and methods used for rPPG with pyVHR toolbox.

Parameters	Description
Window	The number of consecutive video frames processed to estimate the physiological signal.
Holistic	Skin extraction technique that sets the stage for calculating the RGB trace, which is achieved by calculating the average intensity of facial skin colour for each channel separately.
Convexhull	A skin extractor that subtracts the eyes and mouth regions from the rest of the entire face. It offers dependable real-time face and landmark detection and tracking.
CuPy CHROM	A chrominance-based method used to infer the pulse signal from the RGB traces built with the CuPy Python library designed for GPU-accelerated computing with open-source arrays.
Torch CHROM	Built with PyTorch, which is an open-source ML framework that facilitates building, training, and deploying DL models through a dynamic computational graph.
Cupy POS	Plane POS is another method also used to infer the pulse signal from RGB traces, but from a projection plane that is perpendicular to the skin tone built with the CuPy library.

**Table 2:** DL methods implemented.

DL Method	# Layers	Layer (Type)	Output Shape	Param #	Total Params	Trainable Params	Non-Trainable Params
LSTMv1	3	lstm	11,519 × 64	16,896	22,097	22,097	0
		lstm	16	5184			
		dense	1				
LSTMv2	4	lstm	11,519 × 64	16,896	32,465	32,465	0
		lstm	11,519 × 32	12,416			
		lstm	16	3136			
		dense	1	17			
GRUv1	3	gru	11,519 × 64	12,864	16,817	16,817	0
		gru	16	3936			
		dense	1	17			
GRUv2	4	gru	11,519 × 64	12,864	24,689	24,689	0
		gru	11,519 × 32	9408			
		gru	16	2400			
		dense	1	17			
1D-CNNv1	5	conv1d	11,517 × 64	256	1,480,001	1,480,001	0
		max_pooling	5758 × 64	0			
		conv1d	5756 × 32	6167			
		max_pooling	5756 × 32	0			
		flatten	92,096	0			
		dense	16	1,473,552			
		dense	1	17			
1D-CNNv2	7	conv1d	5744 × 512	16896	2,765,441	2,765,441	1792
		max_pooling	1436 × 512	0			
		batch_normalisation	1436 × 512	2048			
		conv1d	1429 × 256	1,048,832			
		max_pooling	357 × 256	0			
		batch_normalisation	357 × 356	1024			
		conv1d	350 × 128	262,272			
		max_pooling	87 × 128	0			
		batch_normalisation	87 × 128	512			
		flatten	11136	0			
		dense	128	1,425,536			
		dropout	128	0			
		dense	64	8256			
		dropout	64	0			
		dense	1	65			
1D-CNNv3	7	conv1d	57,44 × 512	16,896	4,199,169	4,199,169	1792
		max_pooling	1436 × 512	0			
		batch_normalisation	1436 × 512	2048			
		conv1d	1429 × 256	1,048,832			
		max_pooling	357 × 256	0			
		batch_normalisation	357 × 256	1024			
		conv1d	350 × 128	262,272			
		max_pooling	87 × 128	0			
		batch_normalisation	87 × 128	512			
		flatten	11,136	0			
		dense	256	2,851,072			
		dropout	256	0			
		dense	64	16,448			
		dropout	64	0			
		dense	1	65			

**Table 3:** GT-PPG DL models’ results.

DL Method	Domain	Epochs	Accuracy	Precision	Recall	F1-Score	Time [s]
1D-CNNv2	time	100	**83.33%**	83.33%	83.33%	83.33%	28.08
LSTMv1	time	100	79.17%	100.00%	58.33%	73.68%	122.62
GRUv1	time	50	79.17%	81.82%	75.00%	78.26%	60.68
GRUv1	time	100	79.17%	81.82%	75.00%	78.26%	119.67
1D-CNNv3	time	50	79.17%	81.82%	75.00%	78.26%	15.21
LSTMv2	time	50	75.00%	87.50%	58.33%	70.00%	92.04
LSTMv2	time	100	75.00%	87.50%	58.33%	70.00%	182.08
GRUv2	time	50	75.00%	80.00%	66.67%	72.73%	89.58
GRUv2	time	100	75.00%	80.00%	66.67%	72.73%	175.43
1D-CNNv1	time	50	75.00%	87.50%	58.33%	70.00%	5.48
1D-CNNv1	time	100	75.00%	87.50%	58.33%	70.00%	7.62
1D-CNNv3	time	100	75.00%	100.00%	50.00%	66.67%	28.49
1D-CNNv3	frequency	100	75.00%	100.00%	50.00%	66.67%	28.74
LSTMv1	time	50	70.83%	100.00%	41.67%	58.82%	62.28
GRUv1	frequency	100	66.67%	61.11%	91.67%	73.33%	119.30
LSTMv1	frequency	50	62.50%	57.89%	91.67%	70.97%	61.96
GRUv2	frequency	100	62.50%	57.89%	91.67%	70.97%	175.16
LSTMv1	frequency	100	58.33%	55.00%	91.67%	68.75%	122.32
GRUv1	frequency	50	58.33%	56.25%	75.00%	64.29%	60.47
1D-CNNv1	frequency	100	54.17%	52.63%	83.33%	64.52%	7.49
LSTMv2	frequency	100	50.00%	50.00%	100.00%	66.67%	181.87
GRUv2	frequency	50	50.00%	50.00%	8.33%	14.29%	89.23
1D-CNNv1	frequency	50	50.00%	50.00%	100.00%	66.67%	4.24
1D-CNNv2	time	50	50.00%	50.00%	100.00%	66.67%	19.75
1D-CNNv2	frequency	50	50.00%	50.00%	8.33%	14.29%	15.42
1D-CNNv2	frequency	100	50.00%	50.00%	8.33%	14.29%	28.14
1D-CNNv3	frequency	50	50.00%	50.00%	8.33%	14.29%	15.07
LSTMv2	frequency	50	41.67%	41.67%	41.67%	41.67%	92.44

**Table 4:** Comparison of different papers’ results on the UBFC-Phys data.

Work	PPG Method	ML-Method	Accuracy
This work	contact	1D-CNN-MLP	83.33%
	remote		**95.83%**
UBFC-Phys [29]	contact	SVM-linear kernel	73.00%
	remote	SVM-RBF kernel	85.38%
Stress detection using PPG signal and combined deep CNN-MLP network [56]	contact	CNN-MLP	82.00%

**Table 5:** Best DL method results from the rPPG data.

pyVHR Method	DL-Method	Version	Aug.	Domain	Epochs	Accuracy	Precision	Recall	F1-Score	Time
CuPy_CHROM	LSTM	v1	inter	freq	50	83.33%	83.33%	83.33%	83.33%	59.4
		v2	none	freq	100	83.33%	90.00%	75.00%	81.82%	9.8
	GRU	v2	none	freq	50	83.33%	83.33%	83.33%	83.33%	6.3
		v1	none	freq	50	79.17%	76.92%	83.33%	80.00%	4.6
	1D-CNN	**v1**	**wn**	**freq**	**100**	**95.83%**	**100.00%**	**91.67%**	**95.65%**	**7.8**
		**v2**	**inter**	**freq**	**50**	**95.83%**	**100.00%**	**91.67%**	**95.65%**	**14.5**
		v3	inter	time	50	91.67%	100.00%	83.33%	90.91%	15.0
Torch_CHROM	LSTM	v1	inter	freq	50	83.33%	83.33%	83.33%	83.33%	59.6
		v2	none	freq	50	83.33%	90.00%	75.00%	81.82%	6.6
	GRU	v3	wn	freq	100	83.33%	78.57%	91.67%	84.62%	114.7
		v2	none	freq	50	83.33%	83.33%	83.33%	83.33%	6.5
	1D-CNN	**v3**	**inter**	**time**	**50**	**95.83%**	**92.31%**	**100.00%**	**96.00%**	**15.1**
		v2	inter	freq	100	91.67%	100.00%	83.33%	90.91%	27.6
		v1	none	freq	50	87.50%	84.62%	91.67%	88.00%	2.4
CuPy_POS	LSTM	v2	none	freq	50	83.33%	83.33%	83.33%	83.33%	6.0
		v1	inter	time	50	79.17%	76.92%	83.33%	80.00%	59.2
	GRU	v1	none	time	50	83.33%	83.33%	83.33%	83.33%	5.3
		v1	none	time	100	83.33%	83.33%	83.33%	83.33%	9.7
	1D-CNN	v1	inter	time	50	83.33%	83.33%	83.33%	83.33%	14.8
		v3	wn	time	50	83.33%	83.33%	83.33%	83.33%	14.5
		v1	none	freq	50	79.17%	73.33%	91.67%	81.48%	2.1

Aug. (Augmentation), inter (linear interpolation), wn (white noise), and s (seconds).

**Table 6:** Overfitted results of the rPPG data.

pyVHR Method	dl_Method	Aug.	Domain	Epochs	Ac	Pr	Re	F1	Time (s)
CuPy_CHROM	1D-CNNv3	inter	frequency	50	1	1	1	1	14.59
CuPy_CHROM	1D-CNNv3	inter	frequency	100	1	1	1	1	27.00
Torch_CHROM	1D-CNNv2	inter	frequency	50	1	1	1	1	14.44

Aug. (Augmentation), inter (linear interpolation), s (seconds).

## Data Availability

The dataset for this study is publicly available.
